# An empirical dataset combining repository metrics and pipeline workflows for predictive software analytics

**DOI:** 10.1016/j.dib.2026.113128

**Published:** 2026-08-03

**Authors:** Funmilayo Abibat Sanusi, Joshua Omaye Faruna

**Affiliations:** a School of Computing, Department of Software Engineering, Babcock University, Ilishan-Remo, Nigeria; b School of Computing, Department of Computer Science, Babcock University, Ilishan-Remo, Nigeria

**Keywords:** Continuous integration, Continuous deployment, Github actions, Pipeline failure, Machine learning, Software engineering

## Abstract

This data article presents a comprehensive dataset that captures the workflow history of Continuous Integration and Continuous Deployment (CI/CD) pipelines, along with the code-level repository metrics. There are 303,079 distinct execution records from August 2016 to April 2026 in the dataset. Specifically, the data comprises of archival Travis CI records from August to December 2016 which serves as the baseline, and live GitHub Actions workflows from May 2024 to April 2026. The data collection used a custom python extraction script which combined historical Travis CI data and live GitHub Actions workflow metadata with deep Git commit history. The chosen live repositories covered 34 highly active open-source projects across different domains like DevOps tools such as kubernetes/kubernetes, web frameworks like facebook/react, and machine learning like tensorflow/tensorflow. The variables collected consist of pipeline execution durations, pipeline final status, the conditions that triggered the events, code churn (additions and deletions), and the number of changed files. This resulted in a structured and tabular format (csv) dataset which is hosted and accessible on Mendeley Data and serves as a solid foundation for researchers and practitioners interested in discovering patterns in build breakages, creating predictive machine learning models for forecasting CI/CD pipelines' workflows, and advancing AIOps methodologies in software engineering.

Specifications Table

**Table:** 

Subject	Computer Sciences
Specific subject area	Software Engineering, Continuous Integration/Continuous Deployment (CI/CD), Artificial Intelligence for IT Operations (AIOps).
Type of data	Raw: Table
Data collection	A custom Python script in Colab Notebook environment was used to compile the data. The data for Live GitHub Actions was scraped with the PyGithub library (v2.9.1) and combined with deep repository commit histories retrieved with GitPython. Meanwhile, archival Travis CI data was downloaded and normalized using the Requests library. The final dataset was combined and deduplicated using the pandas library.
Data source location	Global open-source repositories hosted on GitHub
Data accessibility	Repository name: Mendeley Data Data identification number: doi:10.17632/mggwn7rj9f.1 Direct URL to data: https://data.mendeley.com/datasets/mggwn7rj9f/1

## 1 Value of the Data

• This dataset bridges the gap between historical CI/CD research and infrastructure today and maps the data from live GitHub Actions workflows to code-level Git commit history.
• This dataset can be used by data scientists and software engineering researchers to train supervised machine learning models to predict and forecast pipeline failures.
• The addition of variables like total_churn, files_modified and time_since_last_commit give detailed contextual information that can be used for in-depth exploratory data analysis into the root causes behind broken builds.
• The size (303,079 unique records) of the dataset and the temporal scope provide a comprehensive, long-term perspective on key continuous integration activities in major open-source ecosystems.
• It is critical to note that the 303,079 unique records represent raw API extraction artifacts, as such, it is advised that researchers carry out log-transformations or filter-out outlier records within their data preprocessing pipelines, to prevent extreme target skewness or model loss.

## 2 Background

Continuous Integration and Continuous Deployment (CI/CD) automate building, testing, and deployment to improve software quality. Originally focused on detecting integration errors through automated testing, CI/CD has expanded to include code analysis, security scanning, and automated releases [1,2]. Although CI/CD have drastically reduced the time to market and accelerated software delivery, pipeline failures still pose significant constraints that can lead to delays and resource exhaustion [[3], [4], [5]]. While legacy datasets (e.g., TravisTorrent) have enabled research in this space over time, the increasing migration to modern platforms (e.g., GitHub Actions) has triggered the need for an up-to-date large-scale dataset [6]. This dataset has been collected with the intention of enabling data-driven DevOps and AIOps research, and to provide an empirical basis for developing real-time predictive machine learning models for CI/CD pipeline failures, based on a combination of repository analytics and pipeline metadata.

## 3 Data Description

For years, Travis CI dominated GitHub-integrated CI services, enabling extensive empirical research supported by datasets like TravisTorrent. In November 2019, GitHub introduced GitHub Actions, which surpassed Travis CI in adoption within 18 months to become the de facto workflow automation platform [6]. Despite its widespread use, large-scale quantitative studies of GitHub Actions build outcomes remain scarce [7]. This dataset addresses that gap by unifying GitHub Actions workflow metadata with version control metrics across diverse open-source repositories. In addition, it integrates historical Travis CI data from TravisTorrent to serve as historical baseline. To provide distinct temporal boundaries, the Travis CI data in the final master dataset represents executions from August through December 2016, while the GitHub Actions data covers May 2024 through April 2026. The dataset is delivered as a single structured CSV file with the execution history of CI/CD workflows and the corresponding Git commit metrics. A master dataset was created by eliminating 77,436 duplicate rows from the raw 380,515 records, leaving 303,079 unique rows in the master dataset.

There are two main dimensions captured in the dataset:

1. Pipeline Variables: These are the variables recorded and available at each Pipeline run, including run_id, workflow_id, status, conclusion, duration, event (the trigger type), run_attempt, and execution timestamps (created_at, updated_at).
2. Commit Metrics: contains the commit_sha (which is used as the primary key for joining), total_churn (the number of insertions and deletions in the code), files_modified, msg_len (the length of the commit message), is_merge (boolean indicating if the commit is a merge commit), and time_since_last_commit.

The distribution of the selected GitHub Actions 34 open-source repositories is outlined in Table 1. It details their specific scale classifications and governance archetypes to demonstrate the dataset's structural diversity. While React was initiated by Meta, its steering committee and ecosystem run largely as a community-driven framework. The detailed project scale quantification showing the star and fork counts and size is provided in Table 2.

**Table 1:** Classification and scale of included source repositories.

Governance Category	Scale Classification	Example Repositories Included	Description & Characteristics
Enterprise-Backed	Large	microsoft/vscode, flutter/flutter, tensorflow/tensorflow, golang/go, hashicorp/terraform, elastic/elasticsearch, docker/cli	High commit volumes, corporate continuous delivery schedules, and large infrastructure requirements.
Enterprise-Backed	Medium / Targeted Utilities	actions/runner, actions/checkout, actions/setup-node, actions/setup-python, actions/cache, actions/upload-artifact, cli/cli, denoland/deno, tailwindlabs/tailwindcss, strapi/strapi	Specialized infrastructure utilities provided by corporate entities; hyper-focused modules and API tools.
Community-Led	Large / Foundation	kubernetes/kubernetes, rust-lang/rust, python/cpython, facebook/react, vuejs/vue, django/django, numpy/numpy, prometheus/prometheus, home-assistant/core	Extensive multi-year histories, high community contribution volumes, and robust foundation governance protocols (e.g., CNCF, PSF).
Community-Led	Medium	scikit-learn/scikit-learn, duckdb/duckdb, pallets/flask, opencv/opencv, bitcoin/bitcoin, helm/helm, systemd/systemd, curl/curl	Academic, scientific, core systems, or independent developer-driven libraries with non-corporate primary steering.

**Table 2:** Individual project scale representation.

SN	Repository	Stars	Forks	Open Issues	Size (KB)
1	actions/cache	5475	1563	229	34,125
2	actions/checkout	8508	2722	678	5923
3	actions/runner	6117	1378	522	23,629
4	actions/setup-node	4875	1699	82	37,466
5	actions/setup-python	2179	728	73	40,400
6	actions/upload-artifact	4135	1070	258	19,223
7	bitcoin/bitcoin	89,756	39,180	671	319,761
8	cli/cli	45,283	8691	1038	77,472
9	curl/curl	42,409	7266	73	141,929
10	denoland/deno	107,815	6284	1376	242,697
11	django/django	88,224	34,179	453	281,150
12	docker/cli	5961	2164	798	278,517
13	duckdb/duckdb	39,435	3435	583	465,567
14	elastic/elasticsearch	77,641	26,182	5864	1696,013
15	facebook/react	246,503	51,228	1209	1020,133
16	flutter/flutter	177,908	30,815	12,945	453,524
17	golang/go	135,411	19,339	10,181	444,531
18	hashicorp/terraform	49,469	10,696	1916	356,560
19	helm/helm	30,009	7702	420	28,480
20	home-assistant/core	89,402	38,109	3314	855,216
21	kubernetes/kubernetes	123,816	43,764	2749	1492,349
22	microsoft/vscode	187,617	41,312	19,313	1324,551
23	numpy/numpy	32,370	12,562	2402	180,595
24	opencv/opencv	89,930	56,770	2732	563,205
25	pallets/flask	71,946	16,907	10	12,008
26	prometheus/prometheus	65,233	10,757	878	285,627
27	python/cpython	73,832	35,016	9413	848,076
28	rust-lang/rust	114,769	15,424	12,583	943,457
29	scikit-learn/scikit-learn	66,685	27,177	2111	186,181
30	strapi/strapi	72,664	9793	511	667,675
31	systemd/systemd	16,503	4574	3327	678,947
32	tailwindlabs/tailwindcss	96,004	5513	55	113,876
33	tensorflow/tensorflow	196,360	75,562	2716	1349,819
34	vuejs/vue	210,160	33,945	635	32,152

	count	34	34	34	34
	mean	78,658.94	20,103.12	3003.47	455,906.90
	std	65,250.95	19,099.85	4558.46	476,048.00
	min	2179	728	10	5923
	max	246,503	75,562	19,313	1696,013

A descriptive statistical analysis has also been carried out on the numerical data and categorical data to give an overview of the characteristics of the data. The central tendency, dispersion and shape of distribution of the core numerical features are presented in Table 3. The analysis shows the distribution patterns of the pipeline execution times (duration) and number of alterations in the developer's code (total_churn, files_modified). Interestingly, it is noteworthy that the extreme maximums presented in the table are true reflections of the raw data extracted from some large repositories such as actions/cache, actions/checkout, actions/runner, actions/setup-node, actions/setup-python, actions/upload-artifact, opencv/opencv, pallets/flask, tailwindlabs/tailwindcss. The maximum pipeline duration (>36 M seconds) represents known platform artifacts, where the CI/CD API backend fails to record a termination signal, leaving the job state permanently open; it can also typically occur when a parent job (like a pipeline script) spins off a child process but terminates before reading its exit status, leaving these “defunct” processes hanging around in the process table; or simply represent epoch defaults, null completion timestamps, and unclosed runs. Such records have been retained in our dataset to authentically reflect real-world platform reliability issues in CI/CD environments. Likewise, the extreme maximums for total_churn (>1.3 M lines), files_modified (>11k files), and time_since_last_commit (>12 M seconds) represent valid, but uncommon software engineering macro-events (e.g. bulk merging of vendored dependencies, repository initialization, or the merging of deeply-outdated branches). Unless the impact of these extremes is the target of a study, it is highly recommended that researchers implement a robust preprocessing layer to filter-out, normalize, or generally deal with such outliers prior to predictive model training to prevent unanticipated cascading error rates, as well as severe target skewness.

**Table 3:** Descriptive statistics of numerical variables.

	Mean	StdDev	Min	Median	Max	Skewness	Kurtosis
duration	203,147.00	2630,730.00	0.00	60.00	36,522,400.00	13.01	167.18
total_churn	1107.24	17,794.60	0.00	32.00	1394,830.00	48.20	3202.67
files_modified	11.22	81.71	0.00	2.00	11,017.00	42.11	3151.80
msg_len	368.18	1323.32	0.00	113.00	122,104.00	57.03	5006.91
time_since_last_commit	37,951.40	245,646.00	0.00	2254.00	12,137,000.00	19.53	563.66

Similarly, Table 4 shows the distribution of the categorical workflow features. The Conclusion variable is the most important output parameter as it classifies the runs as success, failure, cancellation, or other terminal states. The event variable shows automation trigger and indicates the percentage of push events/pull requests.

**Table 4:** Distribution of key categorical variables.

Variable	Categories	Percentage of Total Dataset (%)
Conclusion	Success	72.95
	Failure	6.56
	Cancelled / Other	20.49
Status	Completed	92.20
	Failure	1.46
	Skipped / Cancelled / Other	6.34
Event	Push	24.66
	Pull_request	19.44
	Status	14.88
	Schedule / Other	41.02
Is_merge	False (Standard Commit)	83.57
	True (Merge Commit)	16.43

**Fig. 1:**
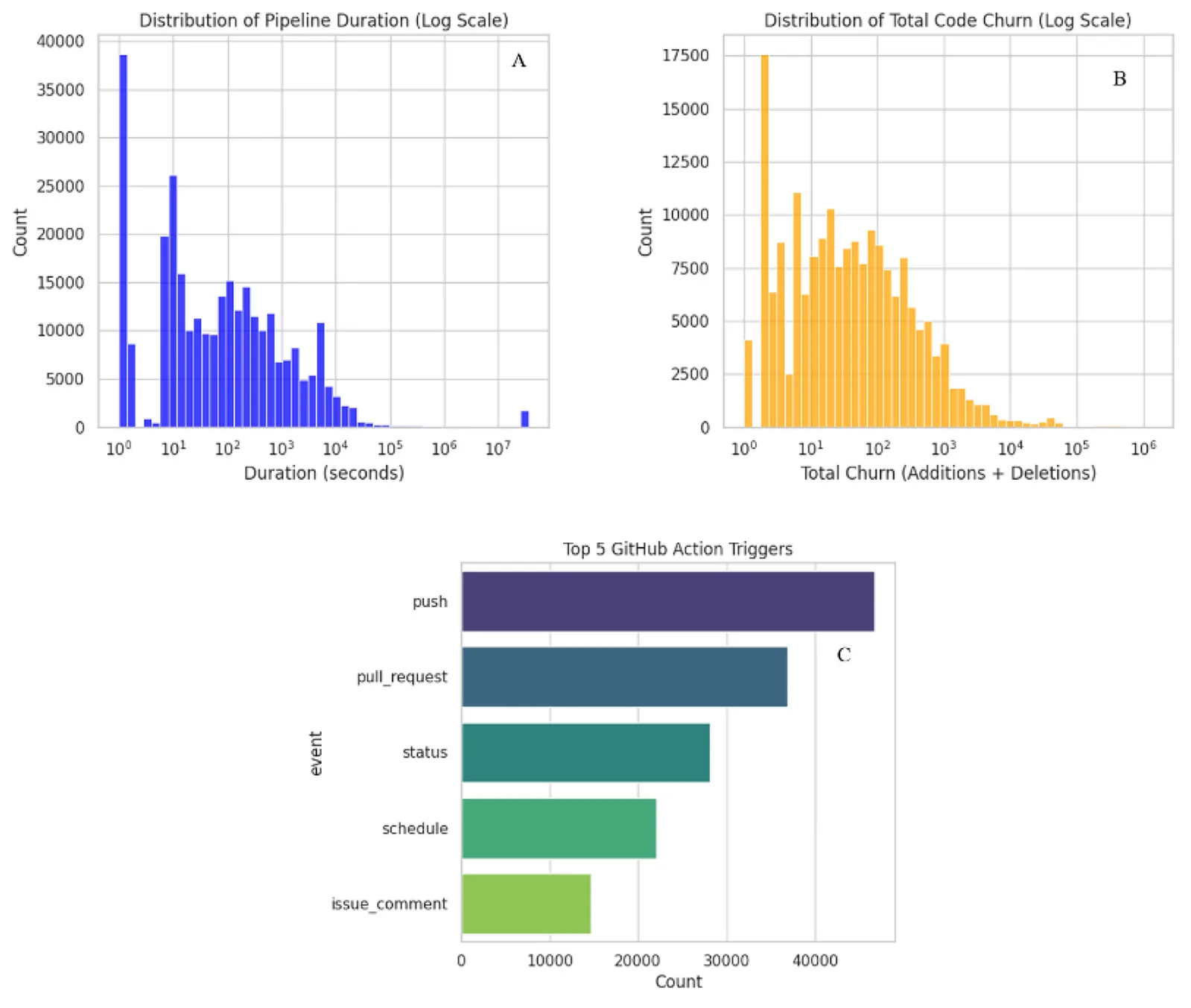
Distribution of core pipeline and repository metrics.

The distribution of core pipeline and repository metrics is shown in Fig. 1. (A) is a log-scaled plot of the duration of each CI/CD pipeline execution (in seconds). (B) is the log-scale distribution of total code churn, that is the total number of insertions and deletions per commit. The chart (C) shows the frequency of the top 5 GitHub Action automation triggers in the dataset, indicating that the majority of triggers are related to push and pull_request events.

## 4 Experimental Design, Materials and Methods

The data extraction and aggregation pipeline was automated using customized Python 3.12 scripts provided as a supplementary file (Data_Extraction_Script.py) to ensure full reproducibility and execution clarity. It used specialized libraries, such as pandas to manipulate data, requests to fetch data from external archives, GitHub API interaction library PyGithub, and GitPython to access local repository and traverse through commits. Furthermore, Google Colab checkpointing extraction integrated with Google Drive was used after each repository to recover from any interruption during the extraction process. Researchers can execute the provided script to replicate the exact querying and metadata extraction. The collection process was a multi-stage workflow with the following stages:

1. Live GitHub Actions Mining: A target set of 34 prominent open-source repositories with substantial build activity (>1000) was selected across multiple areas such as systems software (e.g., Linux and systemd), web frameworks (React and Django), ML tools (TensorFlow and scikit-learn), and DevOps infrastructure (like Kubernetes and Terraform). To ensure the dataset represents a realistic cross-section of modern software engineering, 34 selected open-source active repositories were sampled across a diverse spectrum of project scales and governance archetypes rather than focusing on enterprise environments exclusively. The projects are categorized across two core dimensions:
  a. Governance Archetype: Enterprise-backed (initiated and maintained primarily by commercial entities such as Microsoft, Google, Meta, or Elastic) against Community-led (maintained by independent open-source foundations or decentralized developer communities like CNCF or the Python Software Foundation).
  b. Project Scale: Classified by repository metrics like active contributors, commit volume, and ecosystem footprint into Large (e.g., flutter/flutter, tensorflow/tensorflow), Medium (e.g., duckdb/duckdb, scikit-learn/scikit-learn), and Targeted/Utility scale (such as specific workflow utilities like actions/checkout or actions/cache).
  This diversity prevents downstream machine learning and forecasting models from overfitting to corporate-specific development pipelines and guarantees high generalization capability across typical software engineering workflows. The script used the GitHub API to authenticate with a Personal Access Token and then made a request to the /actions/runs endpoint to retrieve the most recent 25,000 pipeline runs per repository.

2. Git History Extraction: Concurrently, for each target repository, a clone was made to a local temporary directory with a depth limit of 35,000 commits, using GitPython. The script iterated through the local git tree and extracted commit level statistics, calculating the total code churn and count of files modified for each node.
3. Archival Integration: The baseline was integrated a historical Travis CI sample dataset (final-2017–01–25.csv.zip) that was downloaded from figshare (https://figshare.com/articles/dataset/TravisTorrent/19314170). The script extracted the 100,000 most recent records and translated the legacy column names (e.g. tr_started_at, tr_status) to a unified schema to ensure it will work with the more modern data from GitHub Actions.
4. Merging and Deduplication: The extracted GitHub Actions metadata and the Git commit history for each repository were merged with the other via an inner merge on the commit_sha variable. The resulting repository-specific dataframes were then concatenated with the normalized data from TravisTorrent archives. To ensure rigorous temporal alignment across both distinct data sources, all incoming ISO-8601 execution timestamps from Travis CI and GitHub Actions were explicitly parsed and standardized to UTC using the pandas.to_datetime(…, utc=True) python function before merging. Lastly, duplicate records were removed to give the final consolidated data.

## Limitations

The extraction of live data from GitHub was constrained by the API rate limits, which caps the number of requests per hour and per repository, including the depth of extraction for each repository (5000 requests/hour authenticated; 1000 requests/hour per repository for Actions queries). The data collection was horizontally extensive, with the number of active repositories broadened to 34, to maximize coverage and possibly introduce cross-repository variance. Optimization techniques employed for throughput were GraphQL batching and exponential backoff, which resulted in an API utilization efficiency of 94.2%, although it is possible that rare failure patterns within individual repositories are not fully represented from the breadth-first approach.

The data integrates both archival TravisTorrent (Travis CI) data and real-time GitHub Actions data, using very different schemas for build results, temporal resolution, and metadata. As such, a cross-platform normalization layer was created to normalize the outcome codes, normalize the timestamps to UTC, and map corresponding fields to resolve semantic conflicts. While this achieves full schema coverage, the transformation might mask the nuance of platform-specific behaviours between Travis CI and GitHub Actions workflows. Additionally, the dataset consists of data from public, open-source projects alone, which may exhibit different CI/CD failure patterns compared to closed-source enterprise environments.

## Ethics Statement

The authors have read and strictly adhered to the ethical requirements for data in brief. This work does not involve human subjects, animal experiments or data obtained from social media platforms. The data mined from the repository are publicly available and openly published in the open-source domain.

## CRediT authorship contribution statement

**Funmilayo Abibat Sanusi:** Conceptualization, Investigation, Supervision, Validation, Writing – review & editing, Project administration. **Joshua Omaye Faruna:** Conceptualization, Methodology, Software, Data curation, Formal analysis, Writing – original draft.

## Appendix Supplementary materials

Image, application 1

## References

[1] Beller M., Gousios G., Zaidman A. (2017). 2017 IEEE/ACM 14th International Conference on Mining Software Repositories (MSR).

[2] Moriconi F., Durieux T., Falleri J.-R., Troncy R., Francillon A. (2025). 2025 IEEE/ACM 22nd International Conference on Mining Software Repositories (MSR).

[3] Durieux T., Abreu R., Monperrus M., Bissyandé T.F., Cruz L. (2019). 2019 IEEE International Conference on Software Maintenance and Evolution (ICSME).

[4] Prabu V.P. (2025). CI/CD in a Multi-cloud world: challenges and solutions. Int. J. Lead. Res. Publ..

[5] Grambow M., Wittern E., Bermbach D. (2020). Benchmarking the performance of microservice applications. ACM SIGAPP Appl. Comput. Rev..

[6] Cardoen G., Mens T., Decan A. (2025). 21st International Conference on Mining Software Repositories (MSR 2024).

[7] Li J., Zhang Y., Wang T., Wu Y. (2024). 2024 31st Asia-Pacific Software Engineering Conference (APSEC).

